# A Unified Framework for Street-View Panorama Stitching

**DOI:** 10.3390/s17010001

**Published:** 2016-12-22

**Authors:** Li Li, Jian Yao, Renping Xie, Menghan Xia, Wei Zhang

**Affiliations:** 1 School of Remote Sensing and Information Engineering, Wuhan University, Wuhan 430079, China; li.li@whu.edu.cn (L.L.); renping.xie@whu.edu.cn (R.X.); menghx@whu.edu.cn (M.X.); 2 School of Control Science and Engineering, Shandong University, Jinan 250061, China; masakiyo@mail.kitami-it.ac.jp

**Keywords:** panorama stitching, seam line detection, image warping, graph cuts, image parallax, image blending, color correction

## Abstract

In this paper, we propose a unified framework to generate a pleasant and high-quality street-view panorama by stitching multiple panoramic images captured from the cameras mounted on the mobile platform. Our proposed framework is comprised of four major steps: image warping, color correction, optimal seam line detection and image blending. Since the input images are captured without a precisely common projection center from the scenes with the depth differences with respect to the cameras to different extents, such images cannot be precisely aligned in geometry. Therefore, an efficient image warping method based on the dense optical flow field is proposed to greatly suppress the influence of large geometric misalignment at first. Then, to lessen the influence of photometric inconsistencies caused by the illumination variations and different exposure settings, we propose an efficient color correction algorithm via matching extreme points of histograms to greatly decrease color differences between warped images. After that, the optimal seam lines between adjacent input images are detected via the graph cut energy minimization framework. At last, the Laplacian pyramid blending algorithm is applied to further eliminate the stitching artifacts along the optimal seam lines. Experimental results on a large set of challenging street-view panoramic images captured form the real world illustrate that the proposed system is capable of creating high-quality panoramas.

## 1. Introduction

Nowadays, with the development of street-view panoramas, which provide $360^{\circ }$ panoramic views along streets in the real world, the demand for high-quality panoramic images gradually becomes greater. Image stitching is the key technology to produce high-quality panoramic images, which is also an important and classical problem in the fields of photogrammetry [1,2,3,4,5], remote sensing [6,7,8,9] and computer vision [10,11,12,13,14,15], which is widely used to merge multiple aligned images into a single wide-angle composite image as seamlessly as possible.

In an ideally static scene in which both the geometric misalignments and the photometric inconsistencies do not exist or are not obviously visible in overlap regions, the stitched or mosaicked image looks perfect only when the geometric distance criterion is used. However, as we know, most of the street-view panoramic images are captured by a panoramic camera mounted on a mobile platform. Generally, the panoramic camera is comprised of multiple wide-angle or fish-eye cameras whose projection centers are slightly different. Therefore, those images cannot be precisely aligned in geometry; namely, there exist the geometric deviations for corresponding pixels from different images to different extents. In addition, there also exist photometric inconsistencies to different extents in overlap regions between adjacent images due to illumination variations and/or different exposure settings. This paper focuses on creating a visually pleasant street-view panorama by stitching or mosaicking multiple street-view panoramic images among which there may exist the severe geometric misalignments and the strong photometric inconsistencies.

One traditional and efficient way to eliminate the stitching artifacts caused by the large geometric misalignments existing in the input aligned panoramic images is to detect the optimal seam lines that avoid crossing the majority of visually obvious objects and most of the overlap regions with low image similarity and large object dislocation. The optimal seam line detection methods search for the seam lines in overlap regions between images where their intensity or gradient differences are not significant. Based on the optimally-detected seam lines, multiple aligned images can be mosaicked into a single composite image in which the obvious image parallax caused by image misalignments can be magnificently concealed. Many methods [2,3,4,5,6,16,17,18,19] regarded the optimal seam line detection as an energy optimization problem and solved it by minimizing a specially-designed energy function defined to represent the difference between the original images along the seam lines. For these methods, the key ideas concentrate on how to define the effective energy functions and how to guarantee the optimality of the solution. The energy functions are often defined by considering color, gradient and texture and are optimized via different optimization algorithms, e.g., the snake model [20], Dijkstra’s algorithm [21], dynamic programming [22] and graph cuts [23]. Nowadays, the optimal seam line detected by many algorithms can avoid crossing the regions with low image similarity and high object dislocation. In our previous work presented in [19], we proposed an efficient optimal seam line detection algorithm for mosaicking aerial and panoramic images based on the graph cut energy minimization framework. In this paper, we will apply this algorithm to detect the optimal seam lines.

However, when the geometric misalignments are very large, the stitching artifacts perhaps cannot be completely avoided even though the optimal seam lines are detected, especially for street-view panoramic images among which there always exist geometric misalignments to different extents due to those images being captured from scenes with large depth differences by a panoramic camera comprised of multiple wide-angle or fish-eye cameras without a precisely common projection center, which means that the geometric misalignments are different at different positions. Therefore, the large geometric misalignments existing in the input aligned panoramic images should be eliminated as much as possible before finding the optimal seam lines. In this paper, we creatively propose an image warping algorithm based on the optical flow field to reduce the geometric misalignments between input panoramic images. Image warping is a transformation that maps all positions in one image plane to the corresponding ones in another plane [24], which has been popularly applied in many fields of computer vision, such as image morphing [25,26], image retargeting [27,28] and image mosaicking [29,30]. The key technique of image warping is to find the appropriate transformation functions based on the control conditions and then eliminate the distortions between input images. One famous image warping algorithm worked based on thin-plate splines [31] that attempted to minimize the amount of bending in the deformation. They used the radial basis functions with thin-plate splines to find a space deformation defined by control points. However, local non-uniform scaling and shearing possibly occurred in the deformed images. The work in [32] firstly introduced the concept of as-rigid-as-possible transformations, which have the property that both local scaling and shearing are very slight. To produce as-rigid-as-possible deformations, [33] proposed a point-based image deformation technique, which firstly triangulated the input image and then geometrically minimized the distortion associated with each triangle. However, this algorithm needs to triangulate the input image at first, and the results may not be smooth across triangle boundaries. The work in [34] provided an image deformation method based on moving least squares [35] using various classes of linear functions including affine, similarity and rigid transformations. It first found the deformation functions based on the control points or the line segments and then applied the deformation functions on each grid instead of each pixel to reduce the transformation time. At last, it filled the resulting quads using the bilinear interpolation. The work in [36] proposed an image warping algorithm based on radial basis functions, which formulated the image warping problem as the scattered data interpolation problem and used the radial basis functions to construct the interpolation. It aimed at identifying the best radial basis functions for image warping. Our image warping method is similar to this algorithm, but we used the Multilevel B-splines Approximation (MBA) [37] to solve the scattered data interpolation problem. Recently, the b-spline approximation technique has been widely used for image registration [38,39], image morphing, image warping, curve/surface fitting and geometric modeling.

In addition, due to the differences of both the image capturing viewpoints and the camera exposure settings, there are large differences of color and brightness between the warped panoramic images. The large color differences between those images also can cause the stitching artifacts in the last stitched or mosaicked panorama. Furthermore, the large color differences may affect the quality of the seam lines. Therefore, we also need to suppress the color differences between warped images before we apply the optimal seam line detection. Generally, the color correction approaches can be divided into two broad categories according to [40]: parametric and non-parametric. Panoramic approaches assume that the color relationship between images can be described by a certain model. A few noteworthy parametric approaches are described here. The work in [41] proposed a simple linear model to transform the color of the source image to the target image. The transformation matrix was estimated by using the histogram mapping over the overlap regions. The work in [12] applied the gain compensation (i.e., the diagonal model) to reduce color differences between input images. They computed all gains by minimizing an error function, which is the sum of gain normalized intensity errors for all overlapping pixels. The work in [42] also employed the diagonal model for the color and luminance compensation where the correction coefficients were computed as the ratio of the sum of pixel values in the overlap regions. As stated in [43], the linear transformation models can provide a simple yet effective way to transform colors, but they have clear limitations in explaining the complicated nonlinear transformations in the imaging process. Non-parametric approaches can handle this problem well. Non-parametric approaches do not follow any particular model for the color mapping, and most of them use some form of a look-up table to record the mapping of the full range of color levels. As stated in [40], parametric approaches are more effective in extending the color in non-overlap regions without generating gain artifacts, while non-parametric approaches can provide better color matching results. The work in [44] proposed to use the joint histogram of correspondences matched using the SIFT features [45] to correct the color differences. The color mapping function was estimated by using an energy minimization scheme. The work in [46] proposed a color correction approach by using the cumulative color histogram. This method used the cumulative histogram-based mapping to automatically adapt the color of all source images to the reference image. The work in [43] presented a nonlinear and nonparametric color transfer framework that operates in a 3D color space. Based on some control corresponding colors in a given image pair, this method used the probabilistic moving least squares to interpolate the transformation functions for each color. We correct the color differences between two images based on the matched extreme points, which are extracted from the histograms over the overlap regions. Both the Probability Density Functions (PDFs) and Cumulative Distribution Functions (CDFs) are used to find the reliably-matched extreme points. To reduce the gain artifacts in non-overlap regions, we propose to apply the alpha correction method to smooth the transition from non-overlapping regions to overlapping ones.

Although we propose efficient approaches to correct the color differences and detect the optimal seam lines between warped panoramic images, there may also exist some color transitions along the seam lines due to the color differences not being able to be eliminated completely. In order to further conceal these artifacts, the image blending techniques can be further applied along the seam lines. In the last several decades, many image blending algorithms have been proposed to smooth the color differences along the seam lines, such as feathering [47], alpha blending [48], Laplacian pyramid blending [49], Poisson blending [50] and the gradient domain image blending approach [51]. In this paper, we simply applied the Laplacian pyramid blending algorithm [49] to eliminate the stitching artifacts and generate the last pleasant panorama.

**Figure 1:**
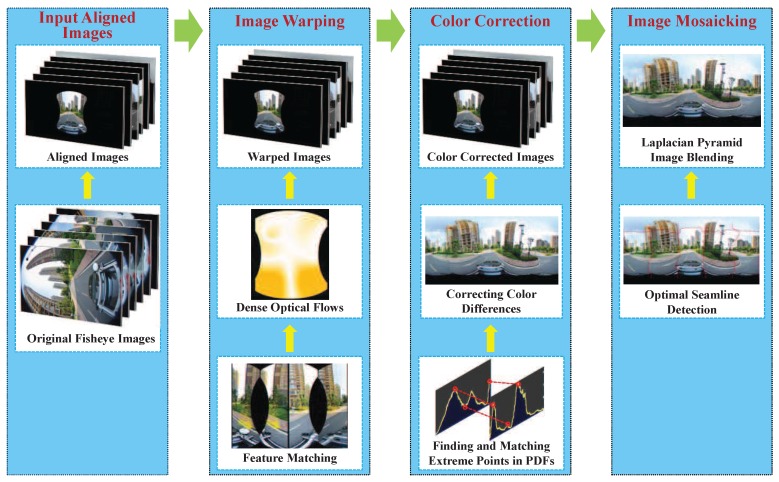
Our proposed unified framework for the street-view panorama stitching system.

In this paper, we propose a unified framework for our developed street-view panorama stitching system, as described in Figure 1. First, multiple original images, which were captured from a single panoramic camera comprised of multiple wide-angle or fish-eye cameras (usually digital SLR cameras) without a precisely common projection center, are fed into our stitching system as the input. Therefore, we will align these input images into a common spherical coordinate system based on the found feature correspondences using the existing open-source library. After that, our proposed image warping method based on the dense optical flow field approximately interpolated from the sparse feature matches, which is detailed described in Section 2, is used to greatly reduce the geometric misalignments. Then, an automatic contrast adjustment and an efficient histogram matching-based color correction approach presented in Section 3 are used to reduce the color differences. Finally, we adopt an efficient seam line detection approach based on the graph cut energy minimization framework to find the optimal seam lines between two overlapped images followed by applying the image blending to eliminate the color transitions along the seam lines. By our proposed unified panorama stitching framework, our system can generate a pleasant street-view panorama as seamlessly as possible by stitching multiple panoramic images from the cameras mounted on the mobile platform. Experimental results on challenging street-view panoramic images are reported in Section 5 followed by the conclusions drawn in Section 6.

## 2. Image Warping

In our developed street-view panorama stitching system, we first check whether all input images are geometrically aligned into a common spherical coordinate system. If not, we will align them by using the open-source library PanoTools (available at http://www.panoramatools.com/), which also serves as the underlying core engine for many image stitching software, such as PTGui (available at http://www.ptgui.com/) and Hugin (available at http://hugin.sourceforge.net/). However, there always exist large geometric misalignments between these aligned images to different extents because those images were captured from scenes with large depth differences by a single panoramic camera comprised of multiple wide-angle or fish-eye cameras without a precisely common projection center. Those geometric misalignments are so large that the stitching artifacts cannot be avoided completely even though the optimal seam lines are detected for image stitching. To ensure the high quality of the last stitched panorama, we propose to apply the image warping technique to eliminate those large geometric misalignments as much as possible. To describe our proposed image warping algorithm more clearly, we first consider a simple case of two aligned images I and $I^{\prime }$ with an overlap. The process of our proposed image warping algorithm is described as follows. Firstly, the corresponding points between two images are found as the control points of image warping, and the sparse optical flows are calculated for those control points. Secondly, the Multilevel B-splines Approximation (MBA) algorithm [37] is used to approximately interpolate the dense optical flows for all integral pixels in the warped image with respect to the original one from the sparse optical flows. Lastly, we warp the two input images based on the dense optical flows, and thus, the geometric misalignments can be greatly lessened. For the case of multiple images, a simple strategy is proposed to first handle the horizontal images and then to deal with the vertical ones.

### 2.1. Feature Point Matching

**Figure 2:**
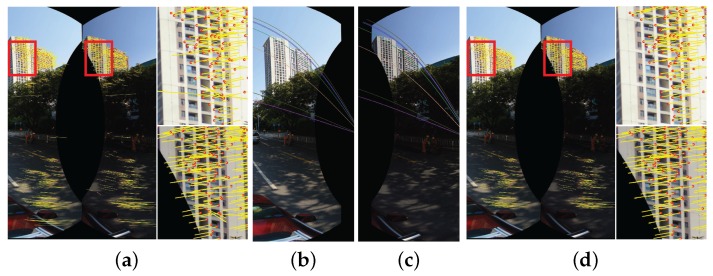
An illustrative example for feature point matching between two aligned panoramic images: (**a**) the point correspondences produced by initial matching; (**b**,**c**) several epipolar lines detected based on the estimated fundamental matrix; (**d**) the last point correspondences after outlier detection. The red circles denote the positions of the matched points in the current image points, and the yellow lines represent the optical flows (i.e., motions) of the matched points in the current image with respect to another image.

To warp two images with large geometric misalignments, we need to find the control points at first. The quality of the warped image mainly depends on the accuracy and densities of control points. In this paper, we apply the feature matching algorithm to robustly find the sparse matching points, namely the control points. The main ideal for feature matching is to first extract local invariant features independently from two images and then characterize them by invariant descriptors. The distance between two descriptor vectors is used to identify candidate matches. However, the nearest neighbors is not always the best match due to occlusion and deformation derived from large viewpoint changes and repeated structures in the scenes. Then, we applied the assumption presented in [52] and the epipolar geometrical constraint to remove the outliers. The major steps of the feature matching include initial matching and outlier detection, which are summarized in Algorithm 1. An example of finding point correspondences between two panoramic images with an overlap is illustrated in Figure 2.
**Algorithm 1** The proposed feature point matching algorithm.***Initial Matching***
(a)Extract and describe two sets of local invariant features from two overlapped images I and $I^{\prime }$ by using the SURF algorithm, respectively;(b)Find the initial point matches $M_{\mathrm{initial}}$ between I and $I^{\prime }$ according to the conditions listed in Section 2.1.1.***Outlier Detection***
(a)Find the neighboring inlier matches $N_{\mathrm{inlier}}\left(f_{p}\right)$ for each match $\langle f_{p},f_{q}^{\prime }\rangle \in M_{\mathrm{initial}}$.(b)Calculate the mean motion $\mu (f_{p})$ and the standard deviation $\sigma (f_{p})$ of all matches in $N_{\mathrm{inlier}}\left(f_{p}\right)$;(c)Identify the outliers according to the criterion defined in Equation (3);(d)Further identify the outliers according to the epipolar geometric constraint.


#### 2.1.1. Initial Matching

Given two adjacent images I and $I^{\prime }$ with an overlap, the local invariant features are extracted and described by the SURF algorithm [53]. Let f=(x,d) be a feature point where $x={\left(x,y\right)}^{⊤}$ denotes the 2D coordinate of this feature point, and d representing its corresponding invariant descriptor vector, and $F={\left\{f_{i}|f_{i}=\left(x_{i},d_{i}\right)\right\}}_{i=1}^{M}$ and $F^{\prime }={\left\{f_{j}^{\prime }|f_{j}^{\prime }=\left(x_{j}^{\prime },d_{j}^{\prime }\right)\right\}}_{j=1}^{N}$ be the feature point sets extracted from I and $I^{\prime }$, respectively, where *M* and *N* denote the numbers of the feature points extracted from I and $I^{\prime }$, respectively. Generally, for one feature point $f_{p}$ in F, the feature point $f_{q}^{\prime }$ with the nearest Euclidean distance $d\left(f_{p},f_{q}^{\prime }\right)=\min_{f_{j}^{\prime }\in F^{\prime }}\left|\right|d_{p}-d_{j}^{\prime }\left|\right|$, which is not larger than a predefined threshold $T_{d}$, can be regarded as the corresponding matching point of $f_{p}$. However, this simple strategy has some drawbacks in the context of feature matching. This mainly because that the distance values between different corresponding pairs may vary in a relatively large range, so any permissive distance threshold $T_{d}$ cannot avoid the appearance of high rate outliers when covering most of the good correspondences. Thus, we propose to modify the matching strategy as follows. In this paper, we accept two feature points $f_{p}$ and $f_{q}^{\prime }$ as a potential match only when they satisfy the following conditions:

- The feature points $f_{p}\in F$ and $f_{q}^{\prime }\in F^{\prime }$ are the nearest neighbors of each other. Namely, for the feature point $f_{p}$, $f_{q}^{\prime }$ is its nearest neighbor in $F^{\prime }$. At the same time, for the feature point $f_{q}^{\prime }$, $f_{p}$ is its nearest neighbor in F.
- The Euclidean descriptor vector distance $d(f_{p},f_{q}^{\prime })$ between two feature points $f_{p}$ and $f_{q}^{\prime }$ is not larger than $T_{d}$, i.e., $d\left(f_{p},f_{q}^{\prime }\right)=∥d_{p}-d_{q}^{\prime }∥\leq T_{d}$.
- We represent the nearest distance between $f_{p}$ and $F^{\prime }$ as $d_{1}\left(f_{p},F^{\prime }\right)=d\left(f_{p},f_{q}^{\prime }\right)=\min_{f_{j}^{\prime }\in F^{\prime }}\left|\right|d_{p}-d_{j}^{\prime }\left|\right|$ and the next distance as $d_{2}\left(f_{p},F^{\prime }\right)=\min_{f_{j}^{\prime }\in F^{\prime },f_{j}^{\prime }\neq f_{q}^{\prime }}\left|\right|d_{p}-d_{j}^{\prime }\left|\right|$, respectively. The distance ratio $r\left(f_{p},F^{\prime }\right)=d_{1}\left(f_{p},F^{\prime }\right)/d_{2}\left(f_{p},F^{\prime }\right)$ should be smaller than the predefined threshold $T_{r}$. Similarly, for the feature point $f_{q}^{\prime }$, the distance ratio $r\left(f_{q}^{\prime },F\right)=d_{1}\left(f_{q}^{\prime },F\right)/d_{2}\left(f_{q}^{\prime },F\right)$ should be smaller than $T_{r}$ as well.

By this matching strategy, we obtain a set of initial matches denoted as $M_{\mathrm{initial}}=\left\{\langle f_{p},f_{q}^{\prime }\rangle |f_{p}\in F,f_{q}^{\prime }\in F^{\prime }\right\}$.

#### 2.1.2. Outlier Detection

After initial matching, there may still exist a few outliers in $M_{\mathrm{initial}}$. Of course, we need to filter out those outliers. According to the assumption proposed by [52] that the matches in a small neighborhood tend to have the consistent location changes (i.e., motions), in this paper, we firstly apply this assumption to identify the outliers. Then, we applied the widely-used constraint of the epipolar geometric to further identify the outliers.

Given a match $\langle f_{p},f_{q}^{\prime }\rangle$, the motions from $f_{p}$ to $f_{q}^{\prime }$ along the horizontal direction and the vertical one are calculated, respectively, as follows:

(1) $$\left\{\begin{array}{c} m_{p}^{\left(x\right)}=x_{q}^{\prime }-x_{p},\\ m_{p}^{\left(y\right)}=y_{q}^{\prime }-y_{p}, \end{array}\right.$$

where $f_{p}={\left(x_{p},y_{p}\right)}^{⊤}$ and $f_{q}^{\prime }={\left(x_{q}^{\prime },y_{q}^{\prime }\right)}^{⊤}$. Thus, the magnitude value of the motion vector ${\left(m_{p}^{\left(x\right)},m_{p}^{\left(y\right)}\right)}^{⊤}$ can be calculated as:

(2) $$m_{p}^{\left(x,y\right)}=\sqrt{{\left(m_{p}^{\left(x\right)}\right)}^{2}+{\left(m_{p}^{\left(y\right)}\right)}^{2}}.$$

Here, we use $m\left(f_{p}\right)={\left(m_{p}^{\left(x\right)},m_{p}^{\left(y\right)},m_{p}^{\left(x,y\right)}\right)}^{⊤}$ to represent all the three motion components of the match $\langle f_{p},f_{q}^{\prime }\rangle$.

At first, we assign the labels of all matches as *Inlier*, namely, for each match $\langle f_{p},f_{q}^{\prime }\rangle \in M_{\mathrm{initial}}$, the label $L(\langle f_{p},f_{q}^{\prime }\rangle )=Inlier$, and then we iteratively find the outliers. For each match $\langle f_{p},f_{q}^{\prime }\rangle \in M_{\mathrm{initial}}$, we find $K_{n}$ ($K_{n}=60$ was used in this paper) neighboring match points of $f_{p}$ from F denoted as the set $N(f_{p})$. Then, we collect all matches whose labels are *Inlier* from $N(f_{p})$ as a new set $N_{\mathrm{inlier}}\left(f_{p}\right)$. If the number of inliers in $N(f_{p})$, namely, the size of $N_{\mathrm{inlier}}\left(f_{p}\right)$, is less than $K_{i}$ ($K_{i}=10$ was used in this paper), we directly label this match as an *Outlier*, namely, $L(\langle f_{p},f_{q}^{\prime }\rangle )=Outlier$, otherwise, we determine whether this match is an inlier by checking whether it has the consistent motion with its neighbors $N_{\mathrm{inlier}}\left(f_{p}\right)$. For each match $\langle f_{m},f_{n}^{\prime }\rangle \in N_{\mathrm{inlier}}\left(f_{p}\right)$, the motion $m(f_{m})$ from $f_{m}$ to $f_{n}^{\prime }$ can be calculated according to both Equations (1) and (2). Then, the mean motion $\mu \left(f_{p}\right)={\left(\mu_{p}^{\left(x\right)},\mu_{p}^{\left(y\right)},\mu_{p}^{\left(x,y\right)}\right)}^{⊤}$ and the standard deviation of all the motions $\sigma \left(f_{p}\right)={\left(\sigma_{k}^{\left(x\right)},\sigma_{k}^{\left(y\right)},\sigma_{k}^{\left(x,y\right)}\right)}^{⊤}$ of all match points in $N_{\mathrm{inlier}}\left(f_{p}\right)$ can be determined easily. According to the following measurement proposed by [52], the label of the match $\langle f_{p},f_{q}^{\prime }\rangle$ can be determined as follows:

(3) $$L\left(\langle f_{p},f_{q}^{\prime }\rangle \right)=\left\{\begin{array}{cc} Inlier, & dist\left(m\left(f_{p}\right),\mu \left(f_{p}\right)\right)\leq \lambda \times \sigma \left(f_{p}\right),\\ Outlier, & \mathrm{Otherwise}, \end{array}\right.$$

where $dist\left(m\left(f_{p}\right),\mu \left(f_{p}\right)\right)=\left|m\left(f_{p}\right)-\mu \left(f_{p}\right)\right|$ denotes the absolute distances in three components between the motion $m(f_{p})$ of the match $\langle f_{p},f_{q}^{\prime }\rangle$ and the mean motion $\mu (f_{p})$ of its neighbor matches. *λ* is a predefined parameter, and we set as λ=3.

After that, we applied the epipolar geometric constraint based on the estimated fundamental matrix to further detect the outliers. The fundamental matrix is estimated by using the RANSAC algorithm. As we known, we cannot estimate the fundamental matrix between two panoramic images which have been projected into the spherical coordinate at first. Therefore, we first project the panoramic images into the perspective plane, and the fundamental matrix is estimated in this plane by using the RANSAC algorithm. The epipolar lines are calculated in the perspective plane at first, and then we re-project them into the spherical coordinate to identify the outliers. At last, we can find all the inliers from $M_{\mathrm{initial}}$ denoted as of the set $M_{\mathrm{inlier}}=\left\{\langle f_{m},f_{n}^{\prime }\rangle |f_{m}\in F,f_{n}^{\prime }\in F^{\prime }\right\}$.

### 2.2. Approximate Interpolation of Dense Optical Flows

Let $\bar{I}$ and ${\bar{I}}^{\prime }$ be the warped images of two adjacent images I and $I^{\prime }$, respectively. The aim of our proposed image warping algorithm is to ensure that the geometric alignments between the warped images $\bar{I}$ and ${\bar{I}}^{\prime }$ become smaller. To achieve this objective, we propose to approximately interpolate the optical flows of all of the integral pixels in $\bar{I}$ with respect to I and all of the integral pixels in ${\bar{I}}^{\prime }$ with respect to $I^{\prime }$ based on the disparity vectors of the reliable point matches with respect to each other as the control points. Firstly, we calculate the disparity vectors $d(x_{m})$ and $d(x_{n}^{\prime })$ of each reliable point match $\langle x_{m},x_{n}^{\prime }\rangle$ in $M_{\mathrm{inlier}}$ from the warped images to the original ones as follows:

(4) $$\left\{\begin{array}{c} d\left(x_{m}\right)=\frac{1}{2}\left(x_{m}-x_{n}^{\prime }\right)=\frac{1}{2}{\left(x_{m}-x_{n}^{\prime },y_{m}-y_{n}^{\prime }\right)}^{⊤},\\ d\left(x_{n}^{\prime }\right)=\frac{1}{2}\left(x_{n}^{\prime }-x_{m}\right)=\frac{1}{2}{\left(x_{n}^{\prime }-x_{m},y_{n}^{\prime }-y_{m}\right)}^{⊤}, \end{array}\right.$$

where $x_{m}={\left(x_{m},y_{m}\right)}^{⊤}$ and $x_{n}^{\prime }={\left(x_{n}^{\prime },y_{n}^{\prime }\right)}^{⊤}$. In this way, we expect to warp the images I and $I^{\prime }$ based on the half offsets of real disparity vectors to reduce the warping distortion.

Secondly, we propose to approximately interpolate the optical flows of all integral pixels in the warped images $\bar{I}$ and ${\bar{I}}^{\prime }$ based on the disparity vectors ${\left\{d\left(x_{m}\right)\right\}}_{x_{m}\in M_{\mathrm{inlier}}}$ and ${\left\{d\left(x_{n}^{\prime }\right)\right\}}_{x_{n}^{\prime }\in M_{\mathrm{inlier}}}$ of the control points ${\left\{x_{m}-d\left(x_{m}\right)\right\}}_{x_{m}\in M_{\mathrm{inlier}}}$ and ${\left\{x_{n}^{\prime }-d\left(x_{n}^{\prime }\right)\right\}}_{x_{n}^{\prime }\in M_{\mathrm{inlier}}}$, respectively. This problem can be formulated as the scattered data interpolation problem. Due to the sparsity of the control points, in this paper, we adapt to apply the Multilevel B-Splines Approximation (MBA) [37] to solve this problem, which has been widely used for image registration, image morphing, image warping, curve/surface fitting and geometric modeling. By this MBA interpolation, we separately interpolate the horizontal and vertical components of optical flows (i.e., disparity vectors) of all the integral pixels in $\bar{I}$ and ${\bar{I}}^{\prime }$, respectively. In this way, we finally obtain the dense optical flows $D\left(\bar{I}\right)={\left\{\tilde{d}\left(p\right)\right\}}_{p\in \bar{I}}$ and $D\left({\bar{I}}^{\prime }\right)={\left\{\tilde{d}\left(p^{\prime }\right)\right\}}_{p^{\prime }\in {\bar{I}}^{\prime }}$ of all of the integral pixels ${\left\{p\right\}}_{p\in I}$ and ${\left\{p^{\prime }\right\}}_{p^{\prime }\in I^{\prime }}$ in the warped images $\bar{I}$ and ${\bar{I}}^{\prime }$ with respect to the original images I and $I^{\prime }$, respectively.

### 2.3. Two Image Warping

Here, we demonstrate how to generate the warped image $\bar{I}$ from the original image I based on the dense optical flows $D(\bar{I})$ of $\bar{I}$ with respect to I, and the generation of the warped image ${\bar{I}}^{\prime }$ is similar. For each pixel $p\in \bar{I}$, we can easily calculate its corresponding 2D position in I based on its approximately interpolated optical flow (i.e., disparity vector) $\tilde{d}\left(p\right)$ as $p+\tilde{d}\left(p\right)$. Then, we use the bilinear interpolation algorithm to interpolate the intensity of the corresponding point $p+\tilde{d}\left(p\right)$ in I as the intensity of the integral pixel $p\in \bar{I}$.

According to the above image warping procedure, we can obtain two warped images from two input panoramic images with the overlap. The geometric misalignments between warped images become smaller than those between the original images after warping correction.

### 2.4. Multiple Image Warping

**Figure 3:**
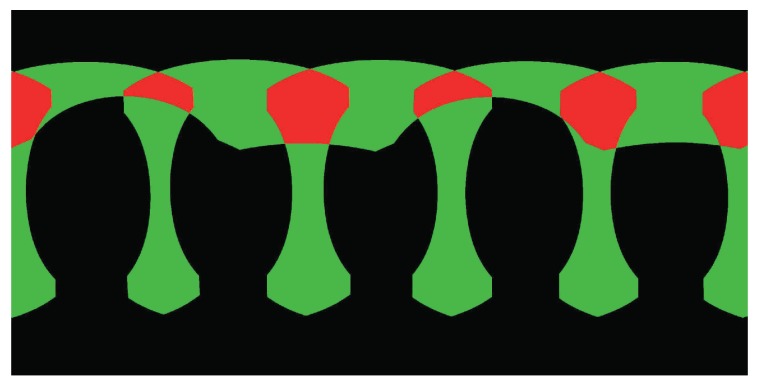
The image overlap regions of six geometrically aligned and warped images in the $360^{\circ }$ street-view panoramic view where the black, the green and the red stand for the no-overlapped, two-overlapped, multi-overlapped image regions, respectively.

**Figure 4:**
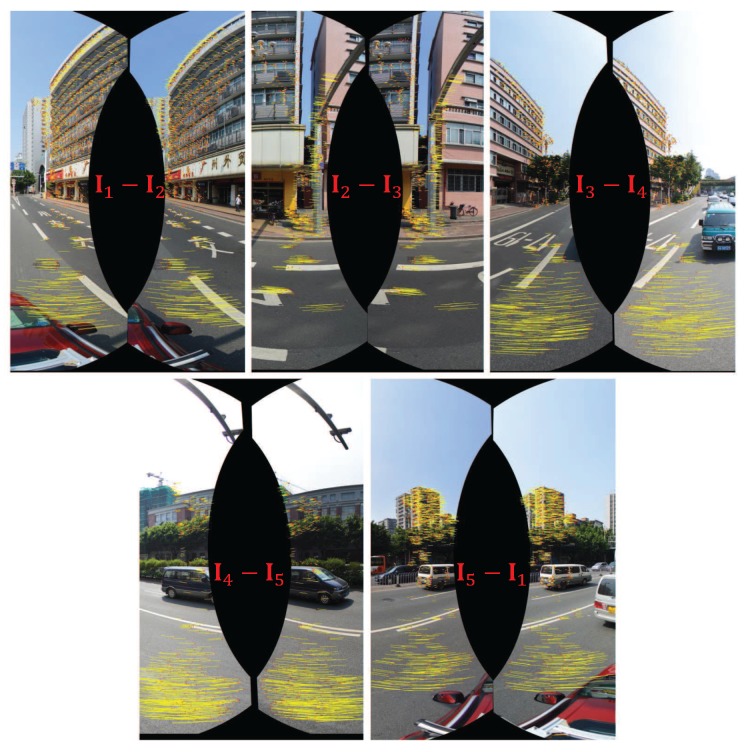
The feature matching results of all five horizontal image pairs in the overlap regions.

**Figure 5:**
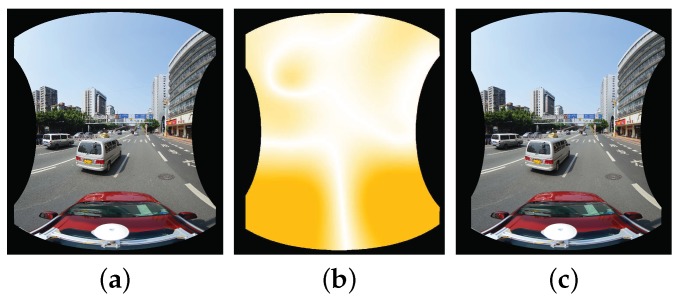
An illustrative example of image warping: (**a**) the original aligned image; (**b**) the dense optical flows approximately interpolated by the Multilevel B-splines Approximation (MBA) algorithm; (**c**) the last warped image. In (**b**), the deeper orange means larger disparity.

Until now, we have introduced how to warp two images based on the optical flows. However, we need to warp multiple input images to generate the last panorama. In the experimental results presented in this paper, the input images are comprised of five horizontal ones and one vertical one, which are represented as $\left(I_{1},I_{2},I_{3},I_{4},I_{5},I_{6}\right)$, whose correspondingly warped images are represented as $\left({\bar{I}}_{1},{\bar{I}}_{2},{\bar{I}}_{3},{\bar{I}}_{4},{\bar{I}}_{5},{\bar{I}}_{6}\right)$, and the overlap relationship of those images is shown in Figure 3. For this particular case, here we will in detail introduce how to warp these six images for producing the last panorama before color correction. Other cases of multiple images can be handled in a similar way. For multiple input panoramic images, we first collect all image pairs according to their overlap relationship, as shown in Figure 3. Obviously, there are five image pairs along the horizontal direction, and one image pair along the vertical direction. We first handle the horizontal image pairs and then deal with the vertical image pair. For each horizontal image pair, we match them one by one by the method presented in Section 2.1, as an illustrative example shown in Figure 4, from which we can find that one horizontal image is overlapped with two adjacent images in the horizontal direction. For example, for the image $I_{1}$, it overlaps with $I_{2}$ and $I_{5}$, respectively, so we need to collect all matching points from these two overlap regions as the control points for warping $I_{1}$. The dense optical flow field of the warped image ${\bar{I}}_{1}$ with respect to the original image $I_{1}$ can be approximately interpolated based on those control points via the MBA algorithm. Therefore, five horizontal warped images ${\bar{I}}_{1}$, ${\bar{I}}_{2}$, ${\bar{I}}_{3}$, ${\bar{I}}_{4}$ and ${\bar{I}}_{5}$ can be generated by warping their corresponding original images according to the method presented in Section 2.3, respectively. Figure 5 shows an example for warping one horizontal image. After that, we generate the bottom blended image $I_{H}$ by blending all horizontal warped images according to the proposed color correction method presented in Section 3 and the adopted image mosaicking strategy described in Section 4. Finally, to produce the last panorama, the top image $I_{6}$ and the horizontal blended image $I_{H}$ will be warped according to those matching points as the control ones.

### 2.5. Summary

In this section, we proposed a new image warping method to eliminate the large geometrical misalignments between adjacent aligned images. This method includes three stages: feature point matching, approximate interpolation of dense optical flows and image warping. In the first stage, we first used the SURF algorithm [53] to find the initial matching points between two images and then applied the assumption presented in [52] and the epipolar constraint to eliminate outliers. The sparse optical flows can be calculated between two matched feature points. In the second stage, we proposed to apply the dense optical flows to simulate the non-rigid deformations between aligned images. The dense optical flows will be interpolated via the MBA algorithm [37] from sparse ones. In the last stage, we proposed to warp the initially aligned images based on the dense optical fields, and we also proposed a strategy to handle multiple image warping. The key contribution of this section is that we proposed to use the optical flows to simulate and eliminate the large non-rigid geometrical misalignments between input aligned street-view images. This method is simple and completed by combining some conventional algorithms, such as SURF, MBA, and so on. However, to the best of the authors’ knowledge, no one has applied this efficient strategy to handle the large geometrical misalignments between street-view panoramic images before color correction and image mosaicking.

## 3. Color Correction

**Figure 6:**
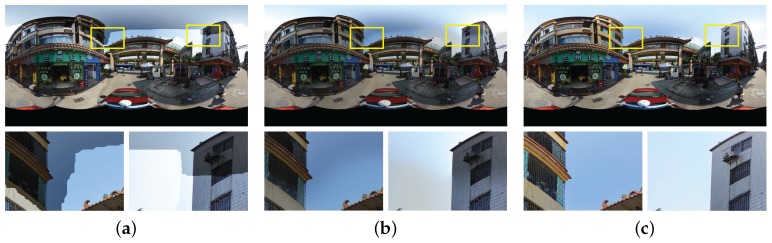
An example of our proposed color correction strategy used to improve the panorama stitching quality before applying the image blending. (**a**) The initial stitched image; (**b**) Only image blending used; (**c**) Image blending after color correction.

The large geometric misalignments can be efficiently eliminated by our proposed image warping algorithm, but there also exist the color differences between the warped images, so the stitching artifacts are still visible. Generally, the image blending technique can solve this easily by smoothing the color along the seam lines. However, it does not work well for input images with very large color differences. The simple image blending may not be able to efficiently conceal the artifacts if we don not magnificently correct color differences between images in advance, which results in low-quality panoramic images, as the illustrative example shown in Figure 6. In addition, the large color differences maybe also affect the quality of the detected seam lines. Thus, in this paper, we propose to reduce the color differences between warped images before the optimal seam lines are detected. Generally, the color differences should be also corrected before the image warping step to ensure the quality of feature matching results. However, our adopted SURF feature matching algorithm is robust enough to the large photometric inconsistencies, so there is no obvious influence on our algorithm if we apply the color correction after image warping.

In this paper, we first apply the automatic contrast adjustment to reduce the brightness differences between images and then propose a novel and efficient color correction algorithm via matching extreme points of intensity histograms to further reduce the color differences. For the overlap image regions between two images, we construct their own Probability Density Functions (PDFs) and Cumulative Distribution Functions (CDFs) with respect to the intensity histograms in the three HSV channels, respectively. One way to eliminate color differences is to ensure that the three CDFs of the overlap regions in the first image in the three HSV channels are approximately the same as those CDFs of the overlap regions in the second image, respectively. Obviously, we can correct the CDFs based on several uniformly-spaced knots, as [54] did. However, due to the existence of geometric misalignments, the scenes presented by two images in the overlap regions are not completely consistent. To solve this problem, we replace the knots by the matched extreme points extracted from the two PDFs. If the number of matched extreme points is not sufficient, we will suitably introduce those uniformly-space knots. At last, the intensities of all of the pixels in the two images are modified afterwards based on the matched extreme points extracted from the PDFs, not only for the pixels in the overlap regions, but also in the non-overlap regions.

### 3.1. Automatic Contrast Adjustment

At first, in order to make sure that multiple images have the similar contrast, which can produce satisfactory blending results, the three RGB channels of individual images are automatically adjusted in contrast. The histograms of a color image are calculated firstly in each of the three RGB channels, respectively. Let I be a single-channel image and $I={\left\{I_{k}\right\}}_{k=1}^{N}$ be a set of one-dimensional sorted intensities of all valid pixels in I in the ascending order, where *N* denotes the total number of valid pixels in I and $I_{k}$ represents the intensity of the *k*-th sorted pixel in I. The minimal and maximal intensities $I_{\min}$ and $I_{\max}$ in I are defined, respectively, as follows:

(5) $$I_{\min}=I_{\left\lceil N\times c\%\right\rceil }\;\mathrm{and}\;I_{\max}=I_{\left\lceil N\times (1-c\%)\right\rceil },$$

where ⌈Δ⌉ denotes the upper integer of a real value Δ and *c* is a small percentage value in the range of (0,50) (c=0.1 was empirically used in this paper), which can be used to skip over a part of the real minimal and maximal intensities due to the fact that these pixels may be caused by noises and information lacking in most cases. The minimal and maximal intensity values of the *R*, *G* and *B* channels of a color image are denoted as $R_{\min}$, $G_{\min}$, $B_{\min}$, $R_{\max}$, $G_{\max}$, and $B_{\max}$, respectively. The minimal and maximal intensity values of the whole color image are defined as $V_{\min}=\min\left(R_{\min},G_{\min},B_{\min}\right)$ and $V_{\max}=\max\left(R_{\max},G_{\max},B_{\max}\right)$, respectively. Therefore, any intensity *I* of the *R*, *G* and *B* channels of a color image will be modified as:

(6) $$I^{\prime }=\left\{\begin{array}{ll} 0, & I\leq V_{\min},\\ 255\times \frac{I-V_{\min}}{V_{\max}-V_{\min}}, & V_{\min}<I<V_{\max},\\ 255, & I\geq V_{\max}. \end{array}\right.$$

In the same way, all of the images to be used for creating a panorama will be automatically adjusted in contrast, which will slightly reduce the brightness differences between images.

### 3.2. Finding Extreme Points

After applying the automatic contrast adjustment on the multiple panoramic images, we propose to further reduce the color differences between panoramic images by matching extreme points of histograms. For the statistic analysis, only valid pixels in the overlap regions between two images are considered. Let A and B be the overlap image regions in two images, respectively. To make a better description of the information hidden behind the image, we convert A and B from the original RGB color space to the HSV color space, respectively. For each channel of A and B, we calculate their PDFs and CDFs, which are denoted as ${\mathrm{PDF}}_{A}$, ${\mathrm{PDF}}_{B}$, ${\mathrm{CDF}}_{A}$ and ${\mathrm{CDF}}_{B}$, respectively.

To robustly find extreme points in both ${\mathrm{PDF}}_{A}$ and ${\mathrm{PDF}}_{B}$, these two PDFs are smoothed first by a Gaussian function to suppress possible noise. The initial local extreme points can be easily obtained from the smoothed ${\mathrm{PDF}}_{A}$ and ${\mathrm{PDF}}_{B}$. In an ideal situation, the extreme points should be uniformly distributed in the color space. However, most of the extreme points are relatively centralized in some cases, which will lead to the information redundancy due to that multiple extreme points being selected out to represent the similar image statistical information. To avoid the situation mentioned above, we further checkout all initial extreme points by the local window suppression. Let ${\left\{L_{A}^{i}\right\}}_{i=1}^{K}$ be the intensities of *K* extreme points ${\left\{P_{A}^{i}\right\}}_{i=1}^{K}$ in ${\mathrm{PDF}}_{A}$, which are sorted in the ascending order. Given an extreme point $P_{A}^{i}$, we generate a neighborhood range $\left[L_{A}^{i}-w,L_{A}^{i}+w\right]$ centered on the corresponding intensity $L_{A}^{i}$ with the size of (2w+1). We set w=2 if not specifically stated in this paper. If there exists more than one extreme points located in this neighborhood range, the extreme point with the highest frequency in ${\mathrm{PDF}}_{A}$ will be retained, and other extreme points will be removed. All initial extreme points are checked in this way, and the retained extreme points are used for the following matching. The final extreme points extracted from ${\mathrm{PDF}}_{A}$ and ${\mathrm{PDF}}_{B}$ are represented as ${\left\{P_{A}^{i}\right\}}_{i=1}^{N_{A}}$ and ${\left\{P_{B}^{j}\right\}}_{j=1}^{N_{B}}$, where $N_{A}$ and $N_{B}$ are the numbers of extreme points in ${\mathrm{PDF}}_{A}$ and ${\mathrm{PDF}}_{B}$, respectively. For each extreme point P, it consists of 4 components according to $P=\{F,L,\hat{C},\overset{ˇ}{C}\}$, where *F* denotes the frequency of this point in PDF, *L* represents the corresponding intensity and $\hat{C}$ and $\overset{ˇ}{C}$ mean the cumulative values of the intensities (L+ε) and (L−ε) in CDF (we set ε=2 if not specifically stated in this paper).

### 3.3. Matching Extreme Points

The extreme points can sufficiently reflect image statistical characteristics. To efficiently adjust the color differences, one way is to ensure that the intensities of corresponding extreme points are the same. Thus, we should match the extreme points firstly. To reliably match these extreme points ${\left\{P_{A}^{i}\right\}}_{i=1}^{N_{A}}$ and ${\left\{P_{B}^{j}\right\}}_{j=1}^{N_{B}}$, we define a cost function to measure the matching similarity of two extreme points $P_{A}^{i}$ and $P_{B}^{j}$ as:

(7) $$Cost\left(P_{A}^{i},P_{B}^{j}\right)=\frac{F_{A}^{i}+F_{B}^{j}}{2F_{\max}}\times \frac{\min(F_{A}^{i},F_{B}^{j})}{\max(F_{A}^{i},F_{B}^{j})}\times \frac{\max({\hat{C}}_{A}^{i}-{\overset{ˇ}{C}}_{A}^{i},{\hat{C}}_{B}^{j}-{\overset{ˇ}{C}}_{B}^{j})}{\max\left({\hat{C}}_{A}^{i},{\hat{C}}_{B}^{j}\right)-\min\left({\overset{ˇ}{C}}_{A}^{i},{\overset{ˇ}{C}}_{B}^{j}\right)},$$

where $F_{\max}$ is the maximal frequency of all of the extreme points in both ${\mathrm{PDF}}_{A}$ and ${\mathrm{PDF}}_{B}$. The above cost function judges the two extreme points from the view of both PDF and CDF. The first term $\frac{F_{A}^{i}+F_{B}^{j}}{2F_{\max}}$ indicates that those possibly matched extreme points with the higher frequencies generate higher costs, which may be peak out first in the following matching selection strategy. The second term $\frac{\min(F_{A}^{i},F_{B}^{j})}{\max(F_{A}^{i},F_{B}^{j})}$ indicates that there are the similar frequencies for two possibly matched extreme points $P_{A}^{i}$ and $P_{B}^{i}$. The last term is applied to ensure that the accumulative values of two possibly matched extreme points $P_{A}^{i}$ and $P_{B}^{i}$ are approximate. From this term, we can find that if the small ranges of cumulative values of two extreme points are similar, the numerator $\max({\hat{C}}_{A}^{i}-{\overset{ˇ}{C}}_{A}^{i},{\hat{C}}_{B}^{j}-{\overset{ˇ}{C}}_{B}^{j})$ is close to the denominator $\max\left({\hat{C}}_{A}^{i},{\hat{C}}_{B}^{j}\right)-\min\left({\overset{ˇ}{C}}_{A}^{i},{\overset{ˇ}{C}}_{B}^{j}\right)$, which results in that this term being close to one. In contrast, if the numerator is smaller and the denominator is larger, this term will approach to zero. In summary, if the frequencies of two extreme points are larger and more similar and the accumulative values of those points are more approximate, their matching cost is bigger. In contrast, it is smaller. The higher the cost function value is, the more likely these two extreme points are matched. Based on this cost definition, a $N_{A}\times N_{B}$ matching cost matrix $M={\left[M_{ij}\right]}_{N_{A}\times N_{B}}$ is created. In order to efficiently eliminate the impossibly matched extreme points, we empirically designed three hard conditions from the view of both PDF and CDF to check whether two extreme points $P_{A}^{i}$ and $P_{B}^{j}$ are possibly matched as follows:

(8) $$\left\{\begin{array}{c} \frac{\min(F_{A}^{i},F_{B}^{j})}{\max(F_{A}^{i},F_{B}^{j})}<\theta_{f},\\ {\overset{ˇ}{C}}_{A}^{i}>{\hat{C}}_{B}^{j}+\theta_{c}\times C_{\max},\\ {\overset{ˇ}{C}}_{B}^{j}>{\hat{C}}_{A}^{i}+\theta_{c}\times C_{\max}, \end{array}\right.$$

where $\theta_{f}$ and $\theta_{c}$ are two empirical thresholds ($\theta_{f}=0.25$ and $\theta_{c}=0.02$ were used in this paper) $C_{\max}$ is the maximal value of CDF, namely the valid pixel number of overlap regions. The matching cost $Cost(P_{A}^{i},P_{B}^{j})$ is set to zero, i.e., $M_{ij}=Cost\left(P_{A}^{i},P_{B}^{j}\right)=0$, if at least one of the above three conditions is not met, namely $P_{A}^{i}$ and $P_{B}^{j}$ are not possibly matched. From the view of PDF, the first condition indicates that the frequencies of the two possibly matched extreme points $P_{A}^{i}$ and $P_{B}^{j}$ should be a relatively small difference. From the view of CDF, the second and third conditions indicate that $P_{A}^{i}$ and $P_{B}^{j}$ are possibly matched if their corresponding CDF values are approximate. According to the above three hard conditions, the matching cost matrix M will be updated, in which all of the zero elements indicate that they are not possibly matched.

Based on the computed matching cost matrix M, we propose an efficient iterative strategy to find the matched extreme points as the following steps:

- Step 1: Finding the highest non-zero cost element $M_{ij}$ from the matrix M and its corresponding extreme points $P_{A}^{i}$ and $P_{B}^{j}$ is selected out as a reliable extreme point match.
- Step 2: Updating the matrix M by removing the *i*-th row and the *j*-th column due to that $P_{A}^{i}$ and $P_{B}^{j}$ have been successfully matched.
- Step 3: Performing the above two steps iteratively until the updated matrix M is empty or there exists no non-zero element in M.

**Figure 7:**
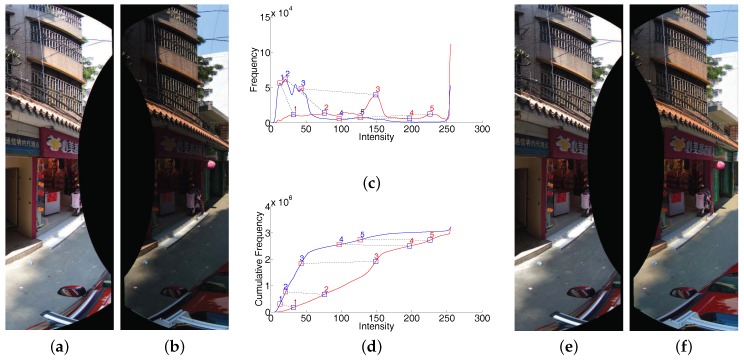
A visual example of our proposed color correction approach: (**a**,**b**) the overlap image regions of the input left and right images, respectively; (**c**,**d**) the curves of PDF and CDF in one channel where the red curves stand for the left image and the blue ones stand for the right image, and the matched peaks are marked by the same number and connected by the black dotted lines; (**e**,**f**) the corrected left and right images, respectively.

By the above iterative strategy, a set of reliable extreme point matches will be found. In Figure 7, we have shown a visual example of our proposed color correction approach. The two input images have large color differences in overlap regions, as shown in Figure 7a,b. We find 5 matched extreme points in the PDF of one channel. Based on those correspondences, the large color differences can be eliminated, as shown in Figure 7e,f. From this example, we can find that our proposed approach can handle the images with large color differences very well.

Sometimes, no match or too few matches can be reliably found via the above matching strategy in the whole CDF range or some relatively large CDF range. In this case, we will introduce more matches with the help of both ${\mathrm{CDF}}_{A}$ and ${\mathrm{CDF}}_{B}$, which are selected from *H* uniformly distributed points ${\left\{C_{A}^{k}\right\}}_{k=1}^{H}$ and ${\left\{C_{B}^{k}\right\}}_{k=1}^{H}$ from ${\mathrm{CDF}}_{A}$ and ${\mathrm{CDF}}_{B}$, respectively, but not from the previously found extreme points. The same number of sampling points in ${\mathrm{CDF}}_{A}$ and ${\mathrm{CDF}}_{B}$ are uniformly selected in accordance with the cumulative density values. In our experiments, the percentages of sampling intervals were used as [0.1, 0.3, 0.5, 0.7, 0.9]. If there exists no extreme point match found in the ranges $\left[C_{A}^{k}-\kappa C_{\max},C_{A}^{k}+\kappa C_{\max}\right]$ and $\left[C_{B}^{k}-\kappa C_{\max},C_{B}^{k}+\kappa C_{\max}\right]$, the current sampling points $C_{A}^{k}$ and $C_{B}^{k}$ will be added into the matching set as a new point match, where *κ* is a given threshold in advance (κ=0.1 was used in this paper).

### 3.4. Correcting Color Difference

**Figure 8:**
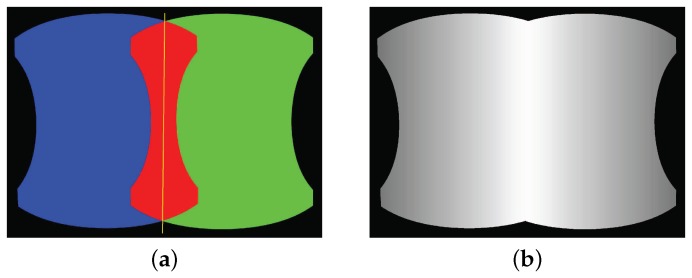
An illustration of the alpha weighting fusion map of two adjacently warped images: (**a**) two overlapped images represented by the blue and green regions, respectively, with the overlap image region marked in red and the center line marked in yellow; (**b**) the normalized alpha weighting fusion map for two images where the brighter regions indicate higher values.

The extracted matching points in the overlap image regions are then applied to correct the intensities of two adjacent images, including the pixels in non-overlap regions. Let ${\left\{Q_{A}^{k}\right\}}_{k=1}^{N}$ and ${\left\{Q_{B}^{k}\right\}}_{k=1}^{N}$ be the final matching points in CDFs in the overlap regions A and B with *N* point matches. Based on the matching results, the intensities of the matching points $Q_{A}^{k}$ and $Q_{B}^{k}$ are modified to $(L_{A}^{k}+L_{B}^{k})/2$ where $L_{A}^{k}$ and $L_{B}^{k}$ denote the intensities of *k*-th match $\left(Q_{A}^{k},Q_{B}^{k}\right)$ in CDFs, respectively. In this way, the intensities of ${\left\{\left(Q_{A}^{k},Q_{B}^{k}\right)\right\}}_{k=1}^{N}$ are corrected to ${\left\{\left({\hat{L}}_{A}^{k},{\hat{L}}_{B}^{k}\right)\right\}}_{k=1}^{N}$, respectively, where ${\hat{L}}_{A}^{k}={\hat{L}}_{B}^{k}=\left(L_{A}^{k}+L_{B}^{k}\right)/2$. Based on these corrections, the intensity of any pixel in both A and B will be adjusted linearly. For example, given a pixel p∈A whose intensity $L_{A}\left(p\right)$ will be linearly corrected as:

(9) $${\hat{L}}_{A}\left(p\right)={\hat{L}}_{A}^{l}+\left(L_{A}\left(p\right)-L_{A}^{l}\right)\frac{{\hat{L}}_{A}^{u}-{\hat{L}}_{A}^{l}}{L_{A}^{u}-L_{A}^{l}},$$

where $L_{A}\left(p\right)\in \left[L_{A}^{u},L_{A}^{l}\right]$, $L_{A}^{u}$ and $L_{A}^{l}$ denote the intensities of two matching points in A that are closest to $L_{A}\left(p\right)$, and the ${\hat{L}}_{A}^{u}$ and ${\hat{L}}_{A}^{l}$ are the corresponding corrected intensities. In order to produce a smooth and gradual transition from non-overlaping regions to overlaping ones, the alpha correction method is conducted as:

(10) $$L_{A}^{\prime }\left(p\right)=\left(1-\alpha \left(p\right)\right)L_{A}\left(p\right)+\alpha \left(p\right){\hat{L}}_{A}\left(p\right),$$

where $L_{A}^{\prime }\left(p\right)$ denotes the finally fused intensity of the pixel p, $L_{A}\left(p\right)$ is the original intensity of the pixel p while ${\hat{L}}_{A}\left(p\right)$ is the corrected intensity of the corresponding pixel based on the above-mentioned correction method, and α(p) is a function that related to the distance between the pixel p and the center line of the overlap image region, which ranges between zero and one as shown in Figure 8, where the smaller the distance to the center line, the larger the *α* is. All the pixels in other images will be processed in the same way.

### 3.5. Summary

In this section, we proposed a new color correction method to reduce the color differences between warped images. This method also includes three stages: automatic contrast adjustment, histogram extreme point detection and matching and color difference correction. In the first stage, we applied the traditional method to adjust the image contrast. This stage can be regarded as the pre-processing. The second stage is the key contribution of the proposed color correction method. In this stage, we proposed a new cost function to evaluate the similarity between two extreme points extracted from two PDFs and then find all matched extreme points iteratively. In the last stage, based on the matched histogram extreme points, the color differences can be corrected easily. The key contribution of this section is that we proposed a new color correction method via matching extreme points of histograms. This method is effective and efficient, which can handle large color differences between two images.

## 4. Image Mosaicking

Although the large geometric misalignments and photometric inconsistencies have been greatly reduced through our proposed image warping and color correction algorithms, respectively, there always exist small geometric misalignments and color differences between adjacent images. To stitch the color corrected panoramic images into the single composite panorama, we also need to find the optimal seam lines in the overlap image regions between warped images to magnificently conceal the parallax. In this paper, the optimal seam lines between color corrected images will be efficiently extracted using the graph cuts-based seam line detection algorithm presented in [19]. For multiple images, we apply the traditional *frame-to-frame optimization* strategy to efficiently find all optimal seam lines. The details of this strategy are described in Section 3.1 of [19]. Furthermore, the Laplacian pyramid blending [49] algorithm will be further applied to eliminate the stitching artifacts caused by small color differences along the seam lines.

## 5. Experimental Results

Extensive experiments on representative street-view panoramic images were conducted to comprehensively evaluate the performance of our proposed unified framework for street-view panorama stitching. In this paper, all used street-view panoramic images were captured from the real world scenes by an integrated multi-camera equipment with six Nikon D7100 cameras of 24 million pixels with wide-angle lenses mounted on a mobile vehicle platform. Six camera images were aligned into a common spherical coordinate system with the image size of 12,000×6000 pixels. Due to that the projection centers of these six cameras are not precisely the same, there always exist large geometrical misalignments at different extents between the adjacently aligned images, especially in the image regions close to the camera centers. The overlap relationship of those six panoramic images is shown in Figure 3. Our algorithms in this paper were implemented with C++ under Windows and tested in a computer with an Intel Core i7-4770 at 3.4 GHz and the 16 GB RAM memory. Due to the limit of pages, more experimental results and analysis are presented at http://cvrs.whu.edu.cn/projects/PanoStitching/. In addition, the stitching framework provided by this paper has been adopted by *IShowChina*(http://www.ishowchina.com/) to product the street-view panoramas for many cities in China. Nowadays, the street-view maps of some cities are already available on *IShowChina*, such as Beijing(http://map.ishowchina.com/index.html#c=110000&s=005200-0-201308190236430390&heading=127pitch=-3zoom=0) and Macao(http://map.ishowchina.com/index.html#c=440400&s=005223-0-201401210342400290&heading=353pitch=10zoom=0). The large set of panoramic images generated by our proposed framework have proved that our framework is robust and effective.

### 5.1. Image Warping

**Figure 9:**
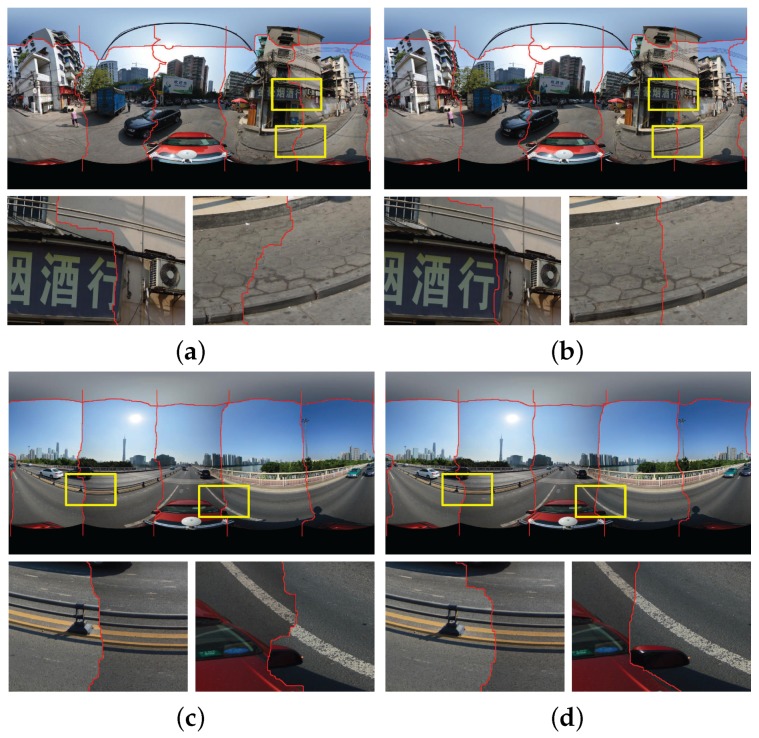
Visual comparison of the stitching results with the optimal seamlines in two groups of six panoramic images when our proposed image warping algorithm was used (Right: (**b**,**d**)) or not (Left: (**a**,**c**), namely, the stitching results of [19]). The red lines stand for the detected optimal seamlines between images.

In this section, we conducted the experiments on two groups of panoramic images to prove the effectiveness and superiority of our proposed image warping algorithm described in Section 2. The panorama stitching results without and with the use of our proposed image warping algorithm in the first group of six panoramic images are shown in Figure 9a,b, respectively. We can find that the whole seamlines in two panoramas cross the similar regions with the high image similarity. However, from the whole stitching results and especially the detailed local regions shown in Figure 9a,b, we observed that the stitching artifacts caused by the geometric dislocation in the panorama, as shown in Figure 9a, stitched without the use of image warping algorithm are more obvious than the panorama, as shown in Figure 9b, stitched with its use. Noticeably, the stitching artifacts caused by geometric dislocation become smaller as expected when the image warping algorithm was applied, as shown in Figure 9b. While not using the image warping algorithm, the geometric dislocation is very large, as shown in Figure 9a. For example, in the first enlarged local region, the seamline crossed the text without the used of image warping, and it avoided crossing the text when the image warping was used. In the second enlarged local region, although two seamlines crossed the road with pavement stairs, we can find that the dislocation is almost invisible in the pavement stairs when the image warping was used, but it is so obvious without the use of the image warping. In the aspect of computational cost, without the use of image warping, our algorithm took around 17.89 s in the above experiment, only the elapsed time in six optimal seamlines detection is included. However, with its use, our algorithm took around 70.93 s consisting of all the elapsed times in the image warping and the optimal seamline detection. From this comparison, we observed that the seamline detection is efficient, but the image warping is relatively time-consuming. This is mainly because that we need to find the inlier matches for all image pairs at first and then interpolate the dense optical flows by MBA for each image, which is time-consuming. But our proposed image warping algorithm can significantly improve the quality of the last stitched panorama.

The comparative experimental results on another group of panoramic images are presented in Figure 9c,d, respectively, and the similar conclusions can be drawn. The computational times of our algorithm without the use of image warping and with its use are 13.19 s and 56.77 s, respectively.

From the above experimental results on two groups of panoramic images, we observed that our proposed image warping algorithm can effectively eliminate the stitching artifacts caused by the geometrical dislocations and can also slightly improve the quality of the found optimal seamlines to some extent.

### 5.2. Color Correction and Image Blending

In this section, we conducted the experiments in two group panoramic images to prove that our proposed color correction algorithm can magnificently reduce the large color differences between the warped images. In addition, we also presented the last panoramas generated by our proposed system and compared them with the open-source software *Enblend* (Available at http://enblend.sourceforge.net/.) which are popularly used to generate the street-view panorama by stitching the registered panoramic images.

**Figure 10:**
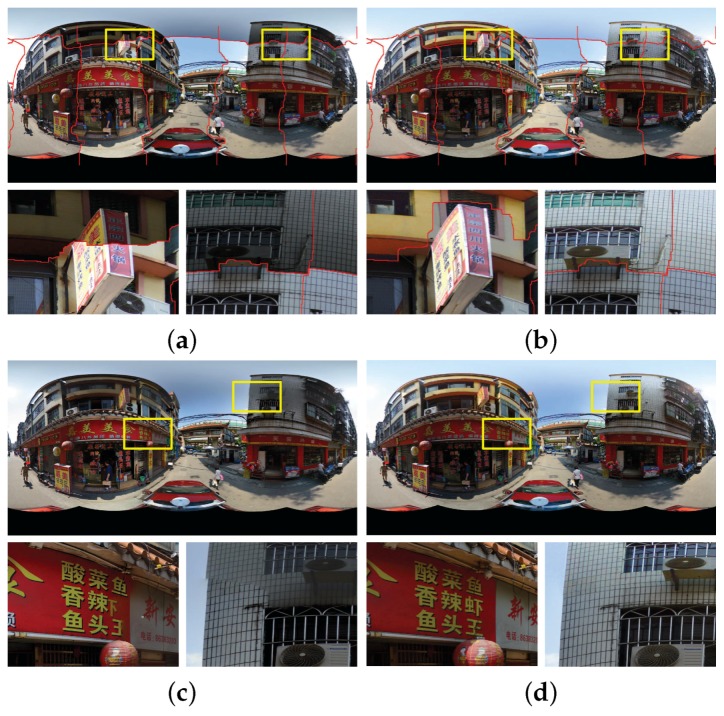
Visual comparison in the first group of six panoramic images: (**a**,**b**) the stitching results with the optimal seamlines when the our proposed color correction was used (**b**) or not (**a**); (**c**,**d**) the last panoramas generated by *Enblend* (**c**) and our proposed stitching system (**d**).

Figure 10 shows the experimental results on the first group of panoramic images. The panorama stitching results without and with the use of color correction are shown in Figure 10a,b, respectively. From the whole stitching results and especially the detailed local regions shown in Figure 10a,b, we can find that color differences between the warped images were significantly reduced and are almost invisible. In addition, the quality of the detected optimal seamlines was improved as expected when the color correction algorithm was used due to that the color differences were greatly reduced before the seamlines were found. For example, the seamline rounded the advertising board instead of crossing it when the color correction algorithm was used, as shown in the detailed image regions in Figure 10a,b. In the aspect of computational cost, without the use of the color correction, our algorithm took around 18.01 s to find all six optimal seamlines. With its use, our algorithm took around 33.52 s to correct the color differences and find the optimal seamlines, means that the color correction algorithm took around 15.51 s. To generate the last panorama, the Laplacian pyramid blending algorithm was further applied, whose generated result is shown in Figure 10d. And in Figure 10c, we also present the last panorama generated by *Enblend*. From the visual comparison, we can observe that our proposed stitching system with image warping and color correction obviously outperforms *Enblend*. Noticeably, the stitching artifacts caused by geometric misalignments and photometric inconsistencies still exist in the panorama generated by *Enblend*, as shown in Figure 10c but they almost disappeared in our produced panorama, as shown in Figure 10d. In the aspect of computational cost, the Laplacian pyramid blending algorithm took 35.56 s.

**Figure 11:**
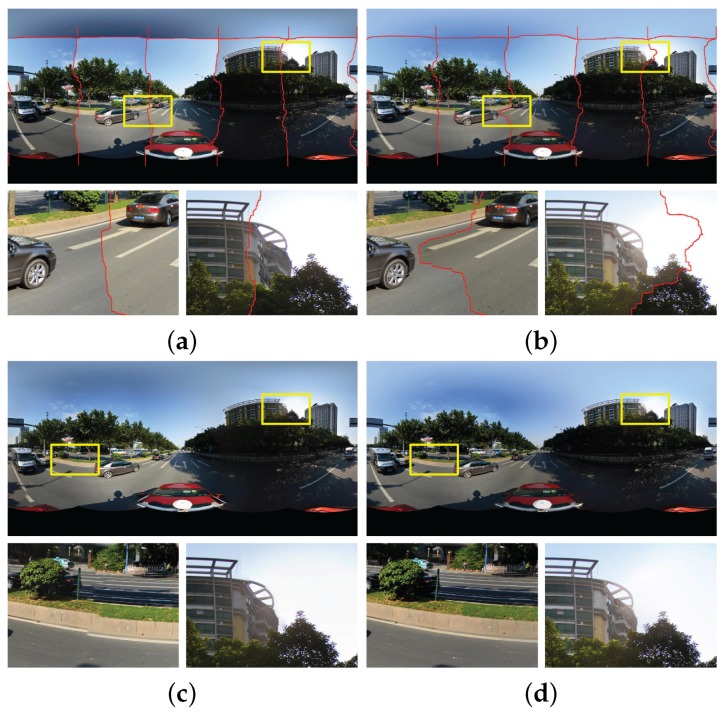
Visual comparison in the second group of six panoramic images: (**a**,**b**) the stitching results with the optimal seamlines when the our proposed color correction was used (**b**) or not (**a**); (**c**,**d**) the last panoramas generated by *Enblend* (**c**) and our proposed stitching system (**d**).

The experimental results on another group of panoramic images are presented in Figure 11 and the similar conclusion can be drawn. The large color differences were greatly reduced by our proposed color correction algorithm, especially in the regions of sky and the tall buildings, and the quality of the detected seamlines was slightly improved to some extent. The seamlines bypass the buildings and the white lane when the color differences were corrected for the warped images. Likewise, the stitching artifacts existed in the panorama produced by *Enblend* disappeared in the panorama generated by our proposed system. The elapsed times in color correction, optimal seamline detection and image blending are 17.33, 13.77 and 35.61 s, respectively.

### 5.3. Image Stitching

**Figure 12:**
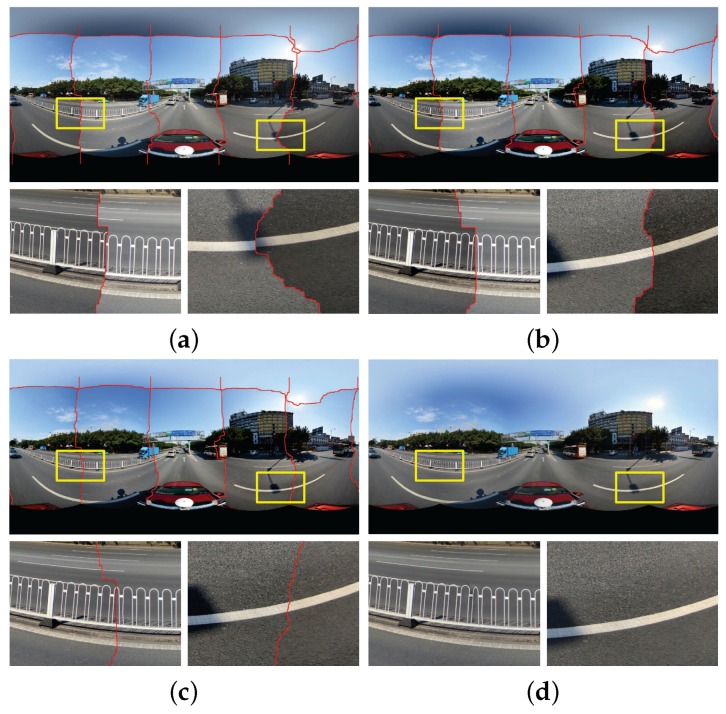
The stitching results with different combination of optimal seamline detection (*S*), image warping (*W*), color correction (*C*) and image blending (*B*) algorithms: (**a**) *S* (the result generated by [19]); (**b**) W+S; (**c**) W+C+S; (**d**) W+C+S+B. The computational times of (**a**–**d**) are 18.00 s, 69.08 s, 86.63 s and 123.68 s, respectively.

To illustrate the effectiveness of our proposed framework for street-view panorama stitching, we presented the last panoramas stitched by different combination of optimal seamline detection (*S*), image warping (*W*), color correction (*C*) and image blending (*B*) algorithms in Figure 12. At first, Figure 12a shows the panorama generated by the optimal seamline detection algorithm presented by [19], from which we can find that there are many stitching artifacts caused by geometric misalignments and photometric inconsistencies in the last stitching image, especially obvious in the detailed local regions. For example, the white lanes on the road were broken due to the large geometric dislocations. In addition, there also exist large color differences along the optimal seamlines. Our proposed image warping and color correction algorithm can eliminate large geometric misalignments and photometric inconsistencies, as shown in Figure 12b,c, respectively. The last blended panorama generated by our proposed system is shown in Figure 12d from which we can observe that the last stitched panoramic image is pleasant and high-quality, which can meet the application requirement of the street-view map.

### 5.4. Comparative Results

At last, to prove that our approach is superior and can generate high-quality panoramas, we compared our proposed approach with another algorithm, the open source library and the open tools. At first, we compared our proposed framework with the Xiong and Pulli’s approach [42] step by step. We used two representative groups of panoramic images for visual comparison. The color differences in the first group of images are relatively small but large in the second group. Because the Xiong and Pulli’s approach has not eliminated the influence of large geometric misalignments between aligned images, so we used the warped images generated by our image warping algorithm as the input ones for comparing two approaches. In addition, their approach applied the Poisson blending algorithm to generate the last blended image, however, our approach used the Laplacian pyramid blending algorithm. To evaluate the last blended panoramas generated by two approaches more fair, we replaced the Poisson blending algorithm in the tested Xiong and Pulli’s approach with the Laplacian pyramid blending algorithm.

**Figure 13:**
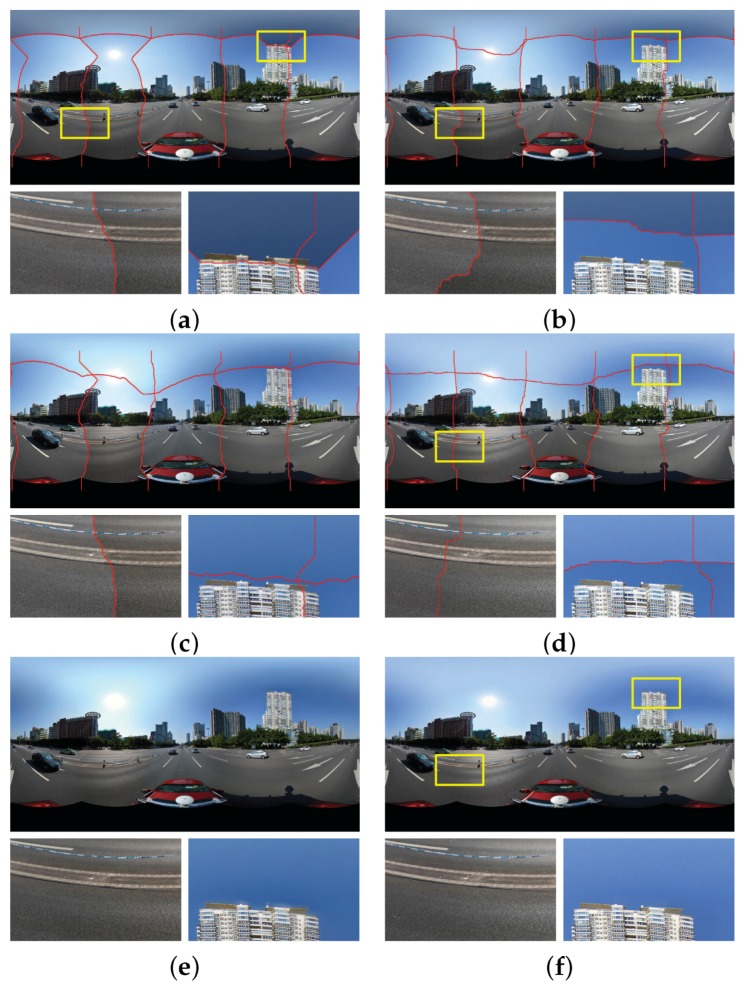
Visual comparison between our approach in the left column and the Xiong and Pulli’s approach in the right column on the first group of images with relatively small color differences: (**a**,**b**) the results without the use of color correction; (**c**,**d**) the results with the use of color correction; (**e**,**f**) the last generated panoramas.

Figure 13 shows the stitching results of the first group of images with relatively small color differences. Figure 13a,b illustrate the stitching results just with the detected seamlines of the Xiong and Pulli’s approach and our approach without the use of color correction, respectively. Figure 13c,d illustrate the stitching results of two approaches with the use of color correction, respectively, from which we observed that both of two approaches can eliminate the small color differences effectively. Figure 13e,f show the last blended panoramas generated by two approaches, respectively, from which we found that there is some petty ghost on the top of the tallest building in the second enlarged region shown in Figure 13e, which disappeared in the panorama generated by our approach, as shown in Figure 13f. This is mainly because the horizontal seamline between bottom and top input images detected by the Xiong and Pulli’s approach is close to this building, as shown in Figure 13c. In conclusion, if the color differences between input images are small, both of two approaches can generate high-quality panoramas.

**Figure 14:**
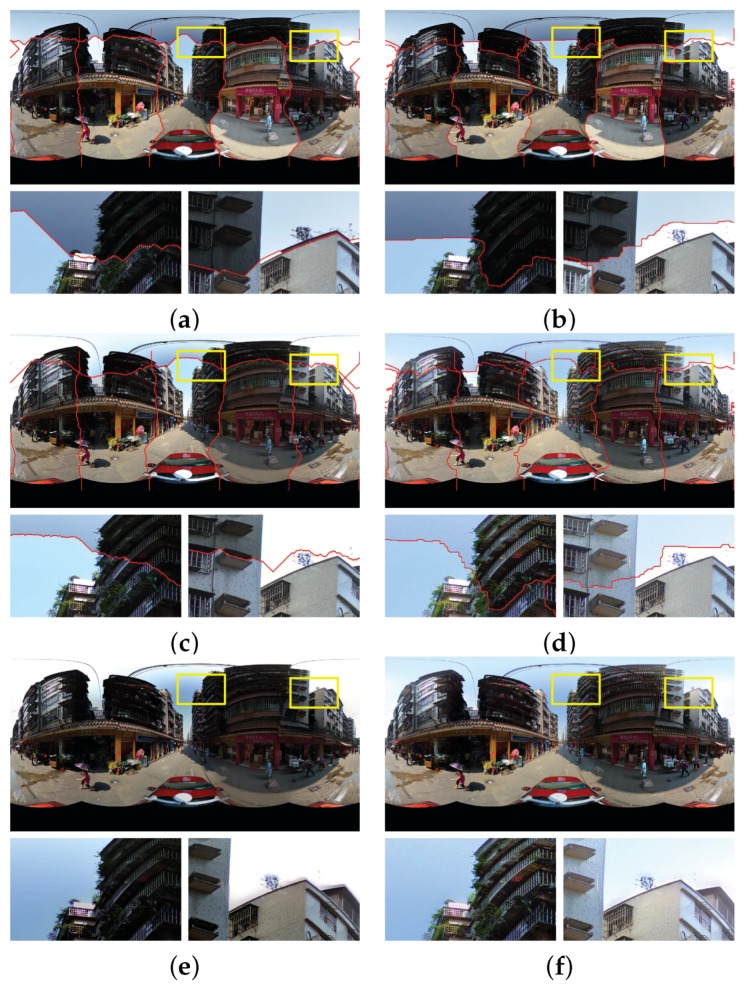
Visual comparison between our approach in the left column and the Xiong and Pulli’s approach in the right column on the second group of images with large color differences: (**a**,**b**) the results without the use of color correction; (**c**,**d**) the results with the use of color correction; (**e**,**f**) the last generated panoramas.

Figure 14 shows the stitching results of the second group of images with very large color differences. Figure 13a,b show the stitching results of the Xiong and Pulli’s approach and our approach without the use of color correction, respectively. Figure 13c,d present the stitching results of two approaches with the use of color correction, respectively. From the visual comparison, we observed that our proposed color correction algorithm obviously outperformed than the algorithm presented in [42], especially obvious in two locally enlarged regions. For example, in the first enlarged region (from left to right), the detected seamline divides the building into two parts, one comes from the top input image which is dark, and another comes from the bottom input image which is relatively lighter. After color correction, the top image is also very dark in the result generated by the Xiong and Pulli’s approach and the color differences along the seamline are also very large. In addition, due to that the top image is too dark, many detailed informations cannot be pleasantly observed. But, in our result, the color of the top image is similar with the bottom one, and more detailed informations of this region can be clearly observed. Figure 13e,f show the last blended panoramas generated by two approaches, respectively. In the second enlarged region of Figure 14e, we found that there are some very obvious ghosts on the top of the building, which disappeared in the panorama generated by our approach, as shown in Figure 14f. In addition, in Figure 14e, the color of top sky regions almost is white, which is not pleasant. However, in the last panorama generated by our approach, the color of those regions is slightly bluish, which is more reasonable and pleasant, as shown in Figure 14f. In conclusion, if the color differences between input images are large, our approach can also generate high-quality panoramas, but the results generated by the Xiong and Pulli’s approach are not so good.

In the aspect of computational times of two approaches, the average times on two groups of images are presented in Table 1, from which we can find that our approach is a litter bit more time-consuming than their approach. This is mainly because their approach applied dynamic programming to detect the optimal seamlines but we used graph cuts, which is more time-consuming than dynamic programming. We also observed that the computation times of our proposed color correction algorithm and their algorithm are 16.83 s and 16.08 s, respectively, which are almost the same. But our color correction algorithm is more effective than their algorithm.

**Figure 15:**
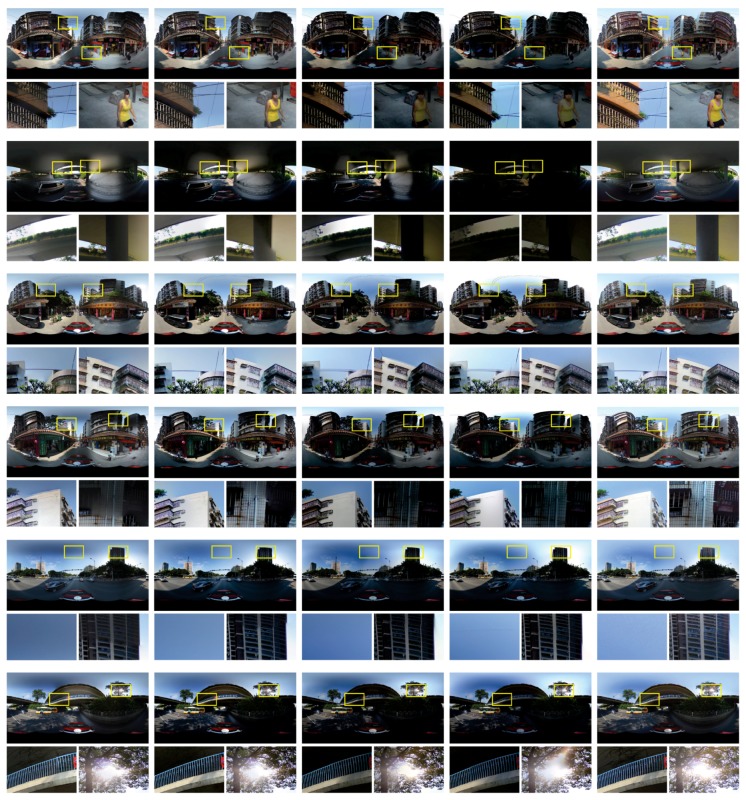
Six groups of panoramic images generated by five different tools or approaches. Form left to right: *Smartblend*, *Enblend*, OpenCV stitching functions, Xiong and Pulli’s approach and our proposed approach. The faces in the first group of images were blurred due to privacy protection.

In addition, we presented more comparative results in Figure 15. We also compared our proposed approach with two widely used open tools *Enblend* and *Smartblend* (Available at http://wiki.panotools.org/SmartBlend), and also with the stitching functions provided by OpenCV (Available at http://opencv.org/). Similar with the experiments presented in Figure 13 and Figure 14, we also used the warped images generated by our image warping algorithm as the input images. From those more visually comparative results, we also can find that our proposed system can generate the best high-quality street-view panoramas.

At last, to strongly prove the superiority of our proposed approach, we applied the Oriented Gradients Image Assessment (OG-IQA) algorithm [55] to quantitatively evaluate the qualities of the final panoramic images stitched by different tools and approaches. Here, we evaluated the panoramic images presented in Figure 13 and Figure 14, and six groups of panoramic images presented in Figure 15. The results of quality assessment are presented in Table 2. In Table 2, we used the Group 1 to Group 6 to represent the panoramic images in the first row to the last row of Figure 15 . From these results, we observed that our approach performed best in most of cases, except of Group 4. But, in Group 4, the quality score of our approach is only litter less than the score of *Enblend*.

## 6. Conclusions

In this paper, we proposed a unified framework for street-view panorama stitching system which is comprised of image warping, color correction, optimal seamline detection and image blending for stitching or mosaicking a set of geometrically aligned street-view panoramic images with large geometric misalignments and photometric inconsistencies into a visual-appealing and informative wide-angle composite image. The contributions in this paper are summarized as follows:

- We creatively proposed a novel image warping method based on the dense optical flows to greatly reduce the large geometric misalignment existed in the input images as much as possible. Experimental results have demonstrated the superiority of our proposed image warping method, which can efficiently and greatly eliminate the influence of the large geometric misalignment.
- We proposed a novel color correction and image blending method to further reduce the color differences between panoramic images based on extreme point matching of histograms of the overlapped image regions of two involved images via both probability density functions and cumulative distribution functions. Experimental results on representative street-view panoramic images have proved that our proposed color correction method is capable of eliminating the large color differences between adjacent images captured in different illumination conditions and/or different exposure settings.
- We proposed a unified framework for street-view panorama stitching system. Even thought there are large geometrical misalignments and photometric inconsistencies in the input aligned images, our system can also generate pleasant and high-quality panoramas. Our proposed framework outperforms the open-source tools *Enblend*, *Smartblend*, OpenCV stitching functions and the approach proposed by [42].

Nevertheless, the proposed system may be improved in the future in the following ways. First, when detecting the optimal seamlines, the superpixel segmentation can be introduced to greatly improve the optimization efficiency by decreasing the number of elements in graph cuts, and the scene understanding or parsing can also be applied in some particular image data. For example, the roads can be detected out for guiding the seamlines. Second, the whole image mosaicking method can be improved to handle more different types of images, not only street-view panoramic ones, but also aerial and oblique ones. At last, the parallel optimization strategy is expected to be developed to more efficiently generate the last panorama.

## Figures and Tables

**Table 1:** The computational times of our proposed approach and the approach proposed in [42].

	Optimal Seamline Detection	Color Correction	Image Blending	#Total
Our Proposed Approach (s)	16.92	16.83	36.60	70.36
Xiong and Pulli’s Approach (s)	13.77	16.08	36.72	66.5815

**Table 2:** The quantitative quality assessments of five tested tools and approaches.

	Figure 13	Figure 14	Group 1	Group 2	Group 3	Group 4	Group 5	Group 6
*Smartblend*	-	-	0.6517	0.6576	0.6011	0.6887	0.6022	0.6498
*Enblend*	-	-	0.7207	0.6773	0.7076	**0.7511**	0.7359	0.7341
OpenCV	-	-	0.6371	0.6279	0.5880	0.6026	0.5522	0.6203
Xiong and Pulli’s Approach	0.5469	0.4693	0.5527	0.4283	0.5306	0.5265	0.6173	0.6075
Our Proposed Approach	**0.7055**	**0.6817**	**0.7248**	**0.7585**	**0.7144**	0.7333	**0.7481**	**0.7469**
